# Author Correction: Enhancing materials property prediction by leveraging computational and experimental data using deep transfer learning

**DOI:** 10.1038/s41467-020-17054-2

**Published:** 2020-07-15

**Authors:** Dipendra Jha, Kamal Choudhary, Francesca Tavazza, Wei-keng Liao, Alok Choudhary, Carelyn Campbell, Ankit Agrawal

**Affiliations:** 1 Department of Electrical and Computer Engineering, Northwestern University, Evanston, IL 60208 USA; 2 Thermodynamics and Kinetics Group, National Institute of Standards and Technology, Gaithersburg, MD 20899 USA

**Keywords:** Inorganic chemistry, Materials chemistry, Density functional theory, Theory and computation

Correction to: *Nature Communications* 10.1038/s41467-019-13297-w, published online 22 November 2019.

The original version of this Article contained some errors due to the presence of duplicates in one of the employed target data sets (EXP), which would have slightly overestimated model accuracy both for the baseline (training from scratch) and transfer learning. Correcting for duplicates in EXP results in small changes in the accuracy numbers, such that a lot of corrections should be done, both in the main text, tables and figures and in the Supplemenary Information file. Please find below a list of the needed corrections.

The last sentence of the abstract originally reads “using an experimental data set of 1,963 observations”. The correct version states ‘1,643’ in place of ‘1,963’.

The last sentence of the abstract originally reads: “the proposed approach yields a mean absolute error (MAE) of 0.06 eV/atom”. The correct version states “0.07 eV/atom” in place of “0.06 eV/atom”.

The second last sentence of the last paragraph of the Introduction originally reads: “and an experimental data set containing 1963 samples from the SGTE Solid SUBstance (SSUB) database”. The correct version states “1,643 samples” in place of “1963 samples”.

The last sentence of the last paragraph of the Introduction originally reads: “in particular, the proposed approach enables us to achieve an MAE of 0.06 eV/atom”. The correct version states “0.07 eV/atom” in place of “0.06 eV/atom”.

The last sentence of the last paragraph of the Introduction originally reads: “against an experimental data set containing 1963 observations”. The correct version states “1,643” in place of “1963”.

The last sentence of the first paragraph of the Results “Data sets” originally reads: “It is composed of 1,963 formation energies at 298.15 K”. The correct version states “1,643” in place of “1,963”.

The last sentence of the second paragraph of the Results “Training from scratch” originally reads: “The impact of training data set is most evident in the case of the experimental data set, where the training data for each fold of the 10-fold cross-validation contains only ~1767 observations and each test (validation) set contains ~196 samples.” The correct version states “~1,479” in place of “~1767” and “~164” in place of “~196”.

The second last sentence of the third paragraph of the Results “Prediction using OQMD-SC model” originally reads: “where the training set in the 10-fold cross-validation contains only ~1770 compositions”. The correct version states: “~1,479” in place of “~1770”.

The second last sentence of the fourth paragraph of the Results “Impact of transfer learning” originally reads: “We believe that an MAE of 0.06 eV/atom...”. The correct version states: “0.07” in place of “0.06”.

The third last sentence of the fifth paragraph of the Results “Impact of training data size on transfer learning” originally reads: “For EXP-SC, we observe a large impact of the training data set size as the MAE decreased from 0.474 eV/atom to 0.124 eV/atom...”. The correct version states “0.436 eV/atom” in place of “0.474 eV/atom”, and “0.133 eV/atom” in place of “0.124 eV/atom”.

The second last sentence of the fifth paragraph of the Results “Impact of training data size on transfer learning” originally reads: “the MAE changes gradually from 0.108 eV/atom to 0.064 eV/atom”. The correct version states “0.106 eV/atom” in place of “0.108 eV/atom”, and “0.071 eV/atom” in place of “0.064 eV/atom”.

The 1st sentence of the 7th paragraph of the Results “Performance on experimental data” originally reads: “by evaluating their performance on the experimental observations containing 1963 samples”. The correct version states “1,643” in place of “1963”.

The fifth last sentence of the last paragraph of the Results “Activation analysis” originally reads: “the ROC curve –0.97 compared with that of ~0.93 using the activations from the model trained from scratch”. The correct version states “0.94” in place of “0.93”.

The second sentence of the caption of Figure 5 originally reads: “The experimental data set contains 1,963 observations”. The correct version should state “1,643” in place of “1,963”.

In Table 1, the numerical values reported for the Experimental data sets are incorrect.

The correct version of Table 1 is:

**Table:** 

Data set	Size	Scratch [SC]	OQMD-SC	Transfer learning [TL]
OQMD	341,000	0.0417 ± 0.0000	–	–
JARVIS	11,050	0.0546 ± 0.0019	0.0821 ± 0.0000	0.0311 ± 0.0012
Materials Project	23,641	0.0326 ± 0.0009	0.1084 ± 0.0000	0.0248 ± 0.0006
Experimental	1,643	0.1325 ± 0.0137	0.1385 ± 0.0000	0.0715 ± 0.0062

which replaces the previous incorrect version:

**Table:** 

Data set	Size	Scratch [SC]	OQMD-SC	Transfer Learning [TL]
OQMD	341,000	0.0417 ± 0.0000	–	–
JARVIS	11,050	0.0546 ± 0.0019	0.0821 ± 0.0000	0.0311 ± 0.0012
Materials Project	23,641	0.0326 ± 0.0009	0.1084 ± 0.0000	0.0248 ± 0.0006
Experimental	1,963	0.1299 ± 0.0136	0.1354 ± 0.0000	0.0642 ± 0.0061

In Table 2, the numerical values reported for the Experimental data sets under Size, Scratch and Transfer Learning are incorrect.

The correct version of Table 2 is:

**Table:** 

Data set	Size	Train:test split ratio	Scratch [SC]	Transfer learning [TL]
OQMD	341,000	8:2	0.0471	–
OQMD	341,000	9:1	0.0437	–
JARVIS	11,050	8:2	0.0593	0.0324
JARVIS	11,050	9:1	0.568	0.0312
Materials Project	23,641	8:2	0.0347	0.0251
Materials Project	23,641	9:1	0.0327	0.0247
Experimental	1,643	8:2	0.1758	0.0762
Experimental	1,643	9:1	0.1484	0.0731

which replaces the previous incorrect version:

**Table:** 

Data set	Size	Train:test split ratio	Scratch [SC]	Transfer learning [TL]
OQMD	341,000	8:2	0.0471	–
OQMD	341,000	9:1	0.0437	–
JARVIS	11,050	8:2	0.0593	0.0324
JARVIS	11,050	9:1	0.568	0.0312
Materials Project	23,641	8:2	0.0347	0.0251
Materials Project	23,641	9:1	0.0327	0.0247
Experimental	1,963	8:2	0.1388	0.0660
Experimental	1,963	9:1	0.1460	0.0608

In Table 3, the numerical values reported for the Experimental data sets are incorrect.

The correct version of Table 3 is:

**Table:** 

Training data set	Test data set	Scratch [SC]	Transfer learning [TL]
OQMD	Experimental	0.1385 ± 0.0000	–
JARVIS	Experimental	0.1915 ± 0.0037	0.1514 ± 0.0030
Materials Project	Experimental	0.1611 ± 0.0020	0.1606 ± 0.0017
Experimental	Experimental	0.1325 ± 0.0137	0.0715 ± 0.0062

which replaces the previous incorrect version:

**Table:** 

Training data set	Test data set	Scratch [SC]	Transfer learning [TL]
OQMD	Experimental	0.1354 ± 0.0000	–
JARVIS	Experimental	0.1911 ± 0.0042	0.1487 ± 0.0027
Materials Project	Experimental	0.1619 ± 0.0020	0.1613 ± 0.0016
Experimental	Experimental	0.1299 ± 0.0136	0.0642 ± 0.0061

The original version of Fig. 3 contains duplicate entries in the Experimental data sets.

The correct version of Fig. 3 is:



which replaces the previous incorrect version:



The original version of Fig. 4 contains duplicate entries in the Experimental data sets.

The correct version of Fig. 4 is:



which replaces the previous incorrect version:



The original version of Fig. 5 contains duplicate entries in the Experimental data sets.

The correct version of Fig. 5 is:



which replaces the previous incorrect version:



The original version of Fig. 6 contains duplicate entries in the Experimental data sets.

The correct version of Fig. 6 is:



which replaces the previous incorrect version:



The above errors have been corrected in both the PDF and HTML versions of the Article.

The original version of the Supplementary Information associated with this Article contained an error in Supplementary Fig. 1, Supplementary Fig. 2 and Supplementary Fig. 3, which contained duplicate entries in the Experimental data set.

The correct version of Supplementary Fig. 1 is:



which replaces the previous incorrect version:



The correct version of Supplementary Fig. 2 is:



which replaces the previous incorrect version:



The correct version of Supplementary Fig. 3 is:



which replaces the previous incorrect version:



The original version of the Supplementary Information associated with this Article contained an error in the third paragraph of the first section of Supplementary Discussion “1. Worst case performance”, which incorrectly reads “0.68 eV/atom and 0.28 eV/atom”: The correct version states “0.61 eV/atom and 0.31 eV/atom” in place of “0.68 eV/atom and 0.28 eV/atom.”

The original version of the Supplementary Information associated with this Article contained an error in the second sentence of the third paragraph of the first section of Supplementary Discussion “1. Worst case performance”, which incorrectly reads “The top 10 elements in the worst predicted set includes O, Ca, Cl, N, F, Te, Sr, B, Pm and I”. The correct version states: “O, N, Ca, Cl, F, P, Mg, Sr, Ni and Ce” in place of “O, Ca, Cl, N, F, Te, Sr, B, Pm and I”.

The original version of the Supplementary Information associated with this Article contained an error in the second sentence of the third paragraph of the first section of Supplementary Discussion “1. Worst case performance”, which incorrectly reads “Similarly, other elements appear in less than 10 materials in the worst set containing 44 samples.” The correct version states “33” in place of “44”.

The original version of the Supplementary Information associated with this Article contained an error in the Supplementary Table 1, in which the numerical values corresponding under the last column (MAE (eV/per atom)) of the Experimental data sets were incorrect.

The HTML has been updated to include a corrected version of the Supplementary Information.

