# Supplementary material for: Enhancing materials property prediction by leveraging computational and experimental data using deep transfer learning
Source: Nat Commun. 2019 Nov 22;10:5316. doi: 10.1038/s41467-019-13297-w (PMC6874674; doi:10.1038/s41467-019-13297-w)
Supplement: Supplementary file 1 — Supplementary Information [file 41467_2019_13297_MOESM1_ESM.pdf]

Enhancing Materials Property Prediction by Leveraging Computational and Experimental Data using Deep Transfer Learning

Jha et al.

Supplementary Discussion

1. Worst Case Performance

We analyzed the aggregated predictions from different ElemNet models trained with and without transfer learning on different datasets. For this we looked at the top 10 elements present in the set of compounds having more than 98th percentile prediction error. Since the size of training dataset has a large impact on performance of a deep learning model, we studied the impact of presence of elements in the training dataset on the prediction errors.

For OQMD-SC (model trained on OQMD from scratch), the 98th percentile error is 0.357 eV/atom, the top 10 elements in the worst predicted set include Pu, O, Np, B, Te, Ru, Sr, Si, Bi and Cs. These elements (except O) are present in only 1,000 to 1,600 materials compounds in OQMD, which is typically less than other elements. Oxygen is present in around 3,200 materials in the dataset, out of which 96 materials are present in the 98th percentile, which is a low proportion.

For models trained on the other three datasets where we apply transfer learning from OQMD-SC, we observe that the 98th percentile drops significantly after transfer learning. For EXP-SC – the model trained from scratch on the experimental dataset, the 98th percentile error are 0.68 eV/atom and 0.28 eV/atom with and without transfer learning respectively. The top 10 elements in the worst predicted set includes O, Ca, Cl, N, F, Te, Sr, B, Pm and I. Oxygen (O) appears in 14 materials in the worst predicted set which is pretty low compared to the total number of materials containing Oxygen in the whole dataset (884). Similarly, other elements appear in less than 10 materials in the worst set containing 44 samples. All these elements exhibit a large reduction in prediction error using EXP-TL due to the use of transfer learning from OQMD-SC; the halides are present in around 1,000 OQMD samples while other elements are heavily present in OQMD. Similarly, for models trained on JARVIS, the 98th percentile drops from 0.225 eV/atom to 0.154 eV/atom after using transfer learning from OQMD-SC; the top 10 elements present in the worst predicted systems being O, F, N, Mn, Cl, Cr, Te, Se, S and Yb. All these elements have significantly low presence in the worst case compared to overall dataset and they all benefit from transfer learning from OQMD-SC, especially the transition metals due to their high presence in OQMD. For the models trained on the Materials Project, the 98th percentile error dropped from 0.141 eV/atom to 0.112 eV/atom after using transfer learning from OQMD-SC; the top 10 elements being O, F, N, S, Cl, C, Cs, Ba, I and B. All elements again benefit heavily from the use of transfer learning from OQMD-SC. Note that we do not have any transition metals in this case.

From this analysis, we observe that O consistently appears at the top element in the worst predicted system for all datasets, which is most likely due to the fact that Oxygen is present in most of the materials present in these datasets. All elements benefit from the use of transfer learning from OQMD-SC, which is trained on OQMD dataset that contains almost all of these elements for a diverse set of compound systems.

2. Comparison against Traditional Machine Learning Approaches

In our previous work [1], we showed how ElemNet outperforms traditional machine learning techniques by automatically capturing the chemical interactions between elements using artificial intelligence. Here, we evaluate traditional machine learning techniques using the same ten-fold cross validation splits as used for ElemNet, as shown in Table 1. We used two types of inputs here- elemental compositions as in ElemNet [1] and 145 physical attributes computed using domain knowledge and intuition [2]. For the case of elemental compositions as inputs, we found that there is no clear winner among the traditional machine learning algorithms. For the case of physical attributes as the input, Random Forest outperforms other traditional machine learning algorithms for all datasets; this is in agreement with the state-of-the-art traditional machine learning based modeling from Ward et al. [2]. For all the datasets, we can observe clearly that the proposed technique of transfer learning from OQMD-SC performs best, outperforming all other approaches.

**Dataset**	**Model**	**Input Type**	**MAE (eV/atom)**
OQMD	KNeighbors	Elemental Compositions	0.1558 ± 0.0000
	Random Forest	Physical Attributes	0.0731 ± 0.0000
	OQMD-SC	Elemental Compositions	**0.0417 ± 0.0000**
JARVIS	Bagging	Elemental Compositions	0.1695 ± 0.0057
	Random Forest	Physical Attributes	0.0858 ± 0.0025
	JAR-SC	Elemental Compositions	0.0546 ± 0.0019
	OQMD-SC	Elemental Compositions	0.0821 ± 0.0000
	JAR-TL	Elemental Compositions	**0.0311 ± 0.0012**
Materials Project	Bagging	Elemental Compositions	0.1401 ± 0.0033
	Random Forest	Physical Attributes	0.0716 ± 0.0016
	MAT-SC	Elemental Compositions	0.0326 ± 0.0009
	OQMD-SC	Elemental Compositions	0.1084 ± 0.0000
	MAT-TL	Elemental Compositions	**0.0248 ± 0.0006**
Experimental	KNeighbors	Elemental Compositions	0.2377 ± 0.0232
	Random Forest	Physical Attributes	0.1227 ± 0.0092
	EXP-SC	Elemental Compositions	0.1299 ± 0.0136
	OQMD-SC	Elemental Compositions	0.1354 ± 0.0000
	EXP-TL	Elemental Compositions	**0.0642 ± 0.0061**

Supplementary Table 1: Benchmarking our deep learning model – ElemNet, against conventional machine learning approaches for formation energy on different datasets. We trained several conventional ML models such as Linear Regression, SGD Regression, Elastic-Net, AdaBoost, Ridge, RBFSVM, Decision Tree, Kernel Ridge, KNeighbors Regression, Bagging and Random Forest; here, we show the results from the best conventional ML model in our comparison study, along with the type of input used, and the resulting mean absolute error (MAE) on the test set. All the models are trained and tested using the same ten-fold cross validation splits as for the ElemNet model. Note that OQMD-SC model is trained using one 9:1 random split.

Supplementary Figures

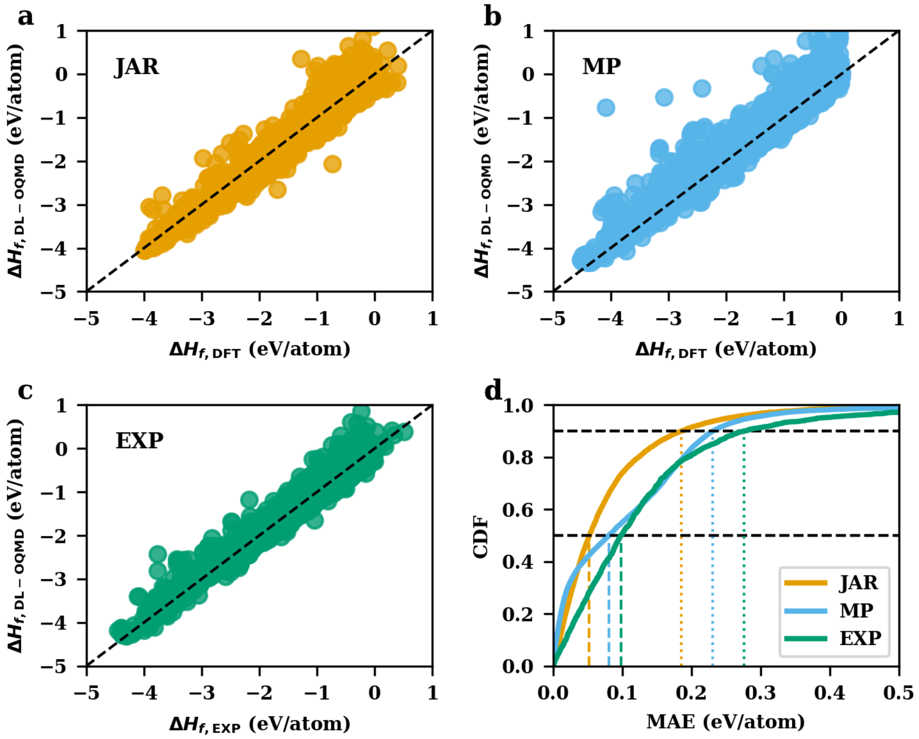

Supplementary Figure 1: Prediction error analysis using OQMD-SC model on the other three datasets. The OQMD-SC model is trained from scratch (with random weight initialization from a uniform distribution) using a 9:1 random split of training and test set from the OQMD. Although OQMD-SC model has low prediction error against the test set from OQMD, the prediction error is high if we compare against other datasets. This is because of the difference in the DFT-computations used in JARIVS (a) and Materials Project (b), and OQMD. Since DFT-computations from the OQMD has an error of around 0.1 eV/atom against experimental observations, this error is inherent in the OQMD-SC model leading to higher prediction errors (c-d).

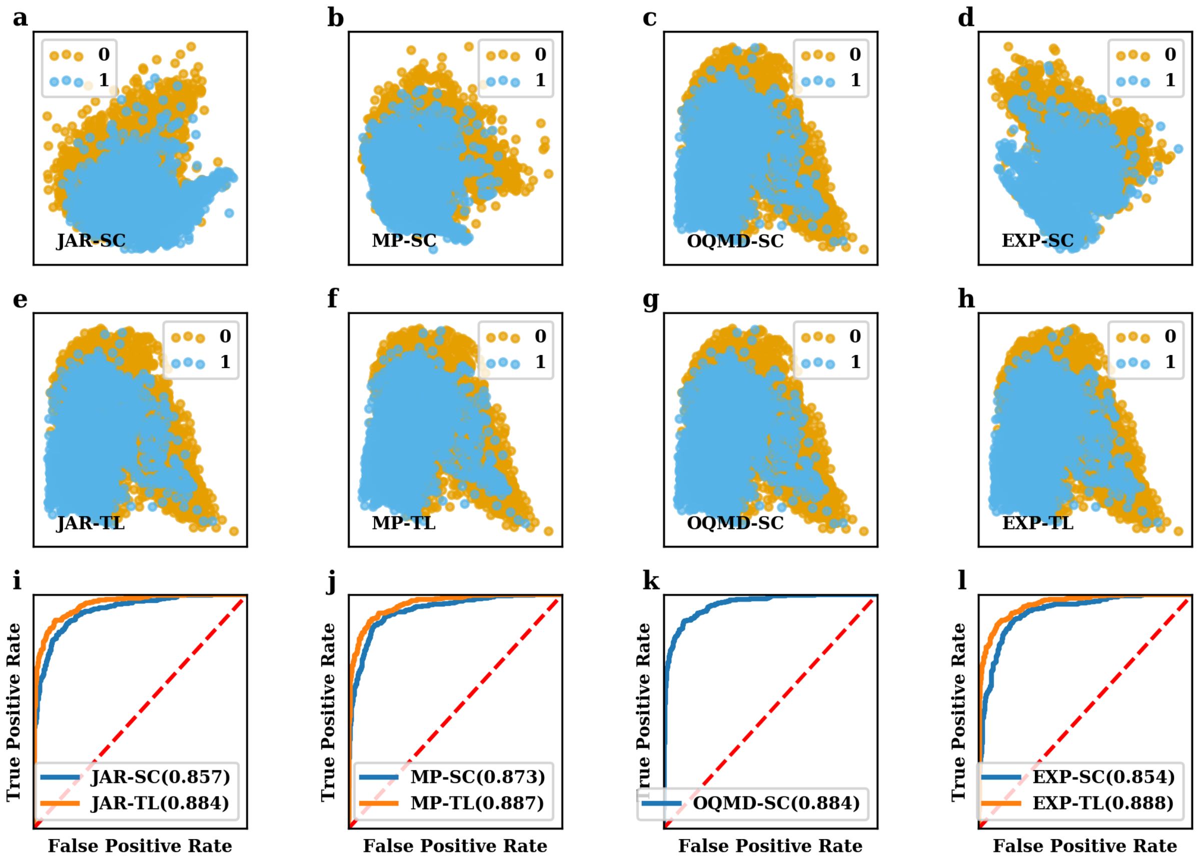

Supplementary Figure 2: Analysis of the activations from the first hidden layer of the ElemNet architecture for the insulator vs metallic class (1 and 0) from JARVIS dataset. The four columns represent the models trained using four different datasets- (a, e and i) JARVIS (JAR), (b, f and j) Materials Project (MP), (c, g and k) OQMD and (d, h and l) the experimental observations (EXP); the first (a-d) and second (e-h) rows represent the models trained from scratch (SC) and using transfer learning (TL), while the third row (i-l) represents the ROC curves from the Logistic Regression model trained using all activations from the same hidden layer (the corresponding AUC values are shown in brackets) on the respective datasets. The scatter plots demonstrate the first two principal components of the activations using principal component analysis (PCA) technique.

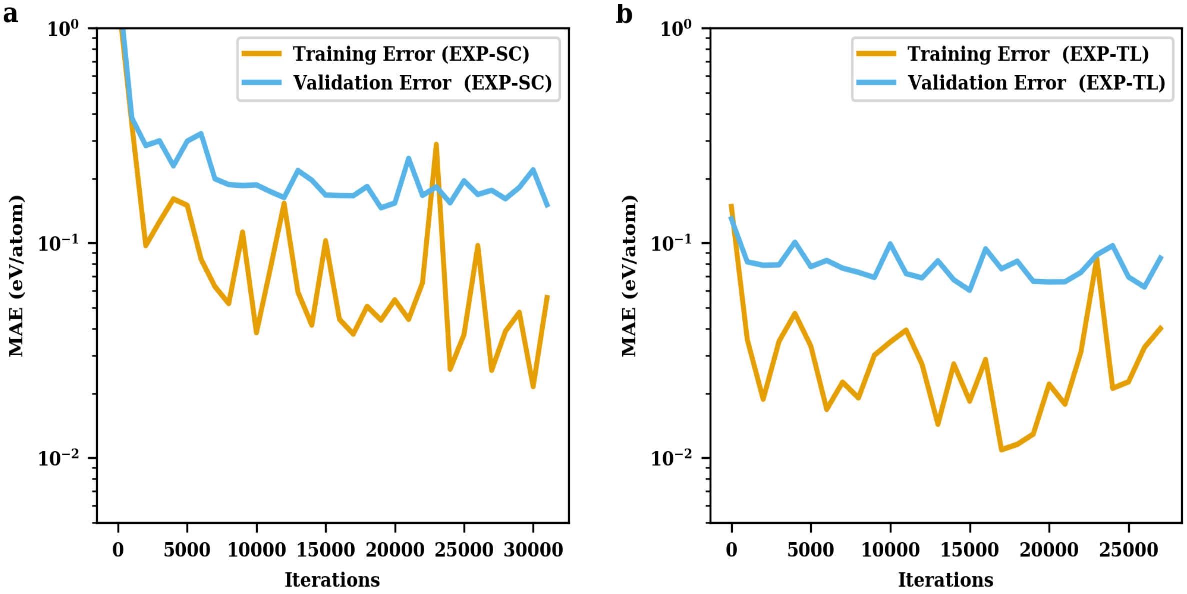

Supplementary Figure 3: Training curve for an ElemNet model trained on experimental dataset containing 1,963 observations from one split of the ten-fold cross validation. We present the training error curve for ElemNet [1] model trained on the experimental dataset from scratch- EXP-SC and using transfer learning from OQMD-SC model- EXP-TL. The training curves show the training and validation error during training the models. When training from scratch in case of EXP-SC (a), the weights are initialized randomly from a uniform distribution; for transfer learning in case of EXP-TL (b), the model weights are initialized from the OQMD-SC model and then fine-tuned using the corresponding dataset. The OQMD-SC model is trained from scratch using a 9:1 random split of training and test set from OQMD. The models were trained using a minibatch size of 64; they were evaluated every 1000 iterations (the training error corresponds to evaluation using current mini-batch, the validation error corresponds to evaluation using whole validation set); since the dataset size is small, the error curve has fewer points and hence not smooth. A patience of 200 minibatch iterations is used for early stopping; the training stops if the validation error does not improve in next 200 minibatch iterations. The error curves are typically used for analyzing the convergence of the model and overfitting to training dataset for proper training.

Supplementary References:

1. Jha, Dipendra, et al. "ElemNet: deep learning the chemistry of materials from only elemental composition." *Scientific reports*8.1 (2018): 17593.
2. Ward, Logan, et al. "A general-purpose machine learning framework for predicting properties of inorganic materials." *npj Computational Materials* 2 (2016): 16028.
